# Phytochemical study on the essential oils of Callitris *glaucophylla* Joy Thomps. & L.A.S. Johnson, and assessment of their antioxidant, anti-enzymatic and allelopathic effects

**DOI:** 10.1016/j.heliyon.2023.e23656

**Published:** 2023-12-13

**Authors:** Oumayma Kochti, Flavio Polito, Lucia Caputo, Khammassi Marwa, Yassine Mabrouk, Lamia Hamrouni, Ismail Amri, Vincenzo De Feo

**Affiliations:** a Laboratory of Biotechnology and Nuclear Technology, National Center of Nuclear Science and Technology, Sidi Thabet, B.P. 72, Ariana, 2020, Tunisia; b Department of Pharmacy, University of Salerno, Via Giovanni Paolo II, 132, 84084, Fisciano, Salerno, Italy; c Laboratory of Management and Valorization of Forest Resources, National Institute of Researches on Rural Engineering, Water and Forests, P.B. 10, Ariana, 2080, Tunisia

**Keywords:** *Callitris glaucophylla*, Phytotoxicity, Antioxidant, Anti-cholinesterase, Anti-Amylase, Anti-Glucosidase

## Abstract

*Callitris glaucophylla* Joy Thomps. & L.A.S. Johnson is a coniferous forest species of the Cupressaceae family native to Australia. This species is rich in essential oils (EOs) but few studies about variability and biological activity of these EOs are available in the literature. The purpose of this study was to evaluate the variability of production of *C. glaucophylla* EOs in relation to the different plant parts (needles, cones and stems) and to investigate their antioxidant, anti-enzymatic and herbicidal properties. EOs were obtained by hydro distillation and analyzed by GC and GC-MS. The antioxidant potential of EOs was assessed by ABTS, FRAP and DPPH assays, their phytotoxic activities were evaluated against germination and shoots and radical growth of *Sinapis arvensis*, *Trifolium campestre, Lepidium sativum* and *Lolium rigidum*. The EOs were evaluated for their possible anti-enzymatic effects with spectrophotometric assay. EOs resulted rich in monoterpenes hydrocarbons (61.04–77.82 %) and oxygenated monoterpenes (19.52–25.26 %). The main compounds were α-pinene as major compound in all plant parts (36.99–59.84 %), 1,8-cineole (19.88 % in stems) and limonene (18.94 % in needles). Herbicidal assays showed that all EOs have remarkable and significant phytotoxicity towards germination, roots, and aerial parts growth of the tested plants, depending on the EO, the doses and tested species. The EOs showed significant free radical scavenging potential and resulted more active against cholinesterases than α-glucosidase and α-amylase. The data obtained constitute an important contribution in selecting and valorizing appropriate forestry tree biomass as sources of antioxidant and phytotoxic molecules for sustainable application in food preservation and weeds control. The activities against the tested enzymes confirmed a possible use of these EOs as natural pesticides.

## 1 Introduction

Studies on the aromatic and medicinal plants are abundant, but the investigation on the forest species remain limited, in particular about the families Cupressaceae and Pinaceae*.* However, plants belonging to these families constitute a great resource, in fact, they occupy large areas, they are generally evergreen, and they can reach more than 20 m in height, which explains the enormous quantities of plant material to be developed and exploited.

Forest trees are known to have several applications, notably as remedies for human diseases and zootechnical applications. Indeed, they contain various secondary metabolites of therapeutic value with many biological effects [1,2]. These metabolites play a pivotal role in the defense against pathogenic parasites and above all in allelopathic interactions [3] This could explain their richness in active molecules with herbicidal, insecticidal and antimicrobial potential that allows numerous agronomic and industrial uses [4]. In addition, forest-derived metabolites may be candidates as alternatives for chemical pesticides [5]. This approach in exploiting the bioactive molecules from forest species can be explained by the fact that forests are accessible and abundant, thus making their development affordable. In addition, the side effects caused by biological molecules are minimal or absent, unlike synthetic molecules [2]. EOs are natural mixtures of volatile secondary metabolites, involved in several biological defense activities [4,5]. Many forest species synthesize EOs [[6], [7], [8]] which can be obtained from needles, leaves, cones, seeds, buds, bark, wood, roots, stems, or fruits [1,6,7,9,10]. The physiological roles of EOs are little known, however, the molecular diversity of the metabolites that they contain gives them multiple biological roles and biological properties [11].

*Callitris glaucophylla* Joy Thomps. & L.A.S. Johnson (the white Cypress pine), belonging to the family Cupressaceae, is a native and widespread tree in Australia. Nowadays, it is widespread in several countries of the world. In 1950, this species was introduced in Tunisia, and it showed a great adaptation to the clime of the country. The plant is known to possess several biological properties. Wilkinson and Cavanagh reported that EOs from Australian native plants including *C. glaucophylla* could be used in cosmetic and pharmaceutical industry as antibacterial agents [12]. Other applications of the EO from *C. glaucophylla* EOs include insecticide [13], antimicrobial and acaricidal activities [14]. *C. glaucophylla* produces secondary metabolites such as guaiol, callitrol, and other substances in its resin that make it highly resistant to decomposition and insect attack [15]. This insect attack resistance is related to the lactones present in the wood oil, which have potential uses as insecticide, antitumor and anti-feeding agent. It was shown that these substances could be obtained from the plant and impregnated with short-lived wood products to enhance protection against termites [15]. Harris and collaborators found that pure *C. glaucophylla* stands could also exclude ground cover [15]. The major components of *C. glaucophylla* EOs are α-pinene, limonene, fenchyl- and bornyl acetate. Similarly, the characterization of the volatile fraction of methanol extracts from two specimens of *C. glaucophylla* showed the presence of the same most components present in the EOs [14].

A study of the different extracts obtained from the hardwood sawdust of *C. Glaucophylla* collected from the Australian forest, revealed their richness in guaiol (40 %), citronellic acid (30 %) and eldanolide. Wood extracts have been investigated for their insecticidal proprierties against *Aedes aegypti* and *Culex annulirostris* larvae and significant insecticidal activities have been displayed by these extracts and even their major components identified [16]. However, the studies on *C. glaucophylla* EOs are limited and all studies are concentrated in Australia and the literature reports were conducted only on the EOs produced by the needles. No chemical and biological investigation on the EOs produced by stems or cones. However, the composition of EOs varies according to tplant's part and the geographical origin of the plant. At light of this context, the aims of this study were: i) to describe the chemical compositions of the EOs of Tunisian *C. glaucophylla* derived from different parts (needles, cones and stems); ii) to study the antioxidant activity of these EOs; to test their phytotoxic activities against germination and growth of some weeds; iv) to evaluate their possible anti enzymatic activity.

## 2 Materials and methods

### 2.1 Plant material

Needles, cones and stems of C*. glaucophylla* were collected on January 2022 from the Korbous region (Nabeul, Tunisia), characterized by a semi-arid Mediterranean climate. Seven samples from several different trees were harvested and then dried on air for 15 days. Seeds of species for herbicidal assays (*Sinapis arvensis* L*. Lolium rigidum* Gaudin*, Trifolium campestre* Schreb and *Lepidium sativum* L.) were harvested from crop fields (Beja, Tunisia). The plants were identified according to the flora of Tunisia at the Laboratory of Forest Ecology of the Institut National de Recherches en Génie Rural, Eaux et Forêts (INRGREF), Tunis by Professor Hamrouni Lamia. Voucher specimena were deposited at the Arboretum of the INRGREF.

### 2.2 Chemical reagent

All chemical reagents used for assays were purchased from Merck, Darmstadt, Germania.

### 2.3 Extraction of the essential oils

The EOs were obtained from *C. glaucophylla* needles, cones and stems by hydro-distillation for 3 h. The EOs were dried and stored in sealed vials at 4 °C before analysis and bioassay studies.

The yield was calculated considering dry weight of the sample by formula (1):

(1) $$\text{Oil}\;\text{yield}\;\left(\%,W/W\right)=\frac{\text{weight}\;\text{of}\;\text{essential}\;\text{oils}\;\left(g\right)}{\text{dried}\;\text{weight}\;\text{of}\;\text{plant}\;\text{material}\;\left(g\right)}*100$$

### 2.4 GC-FID and GC/MS analyses and identification of the essential oil components

The composition of the EOs was analyzed using GC and GC-MS methods used by Polito and coworkers [17]. Most of the compound were identified comparing their Kovats indices (Ki) with the literature [[18], [19], [20], [21]] and withthe mass spectra of pure compounds available in our laboratory. The Kovats indices were calculated in relation to a series of n-alkanes (C_10_–C_35_), under the same conditions. In some case, the identification was corroborated by co-injection with standard samples.

### 2.5 Antioxidant activity

#### 2.5.1 2,2 -Diphenyl-1-picrylhydrazyl (DPPH) assay

Free radical scavenging activity of *C. glaucophylla* oils was assessed using the DPPH assay, according to the method described by Ud-Daula et al. [22] with following modifications. The assay was carried out in cuvettes by adding 25 μL of different EOs concentrations to 975 μL of a DPPH solution (6 × 10^−5^ M). DPPH solution was used as a negative control. Cuvettes were read at 515 nm using spectrophotometer Thermo scientific Multiskan GO (Thermo Fischer Scientific, Vantaa, Finland) after incubation of 45 min. The scavenging effect on the DPPH radical of the samples could be calculated as IC_50_ values, which are the quantity of EOs necessary to inhibit radical DPPH activity by 50 %. Trolox (6-hydroxy-2,5,7,8-tetramethylchroman-2-carboxylic acid) was used as a reference standard.

#### 2.5.2 ABTS^•+^ free radical scavenging activity

The ABTS^**•+**^ radical assay was carried out according to protocol reported by Ud-Daula et al. [22] with minor modifications. The mix of ABTS and potassium persulfate solution with a final concentration of 7 and 2.45 mM, respectively, was incubated in the dark at 25 °C for 16 h before use to produce the radical ABTS (ABTS+). The mix was diluted with distillated water to an OD of 0.800 at 734 nm. The results were expressed as IC_50_ values, which are the amount of EOs necessary to reduce radical ABTS activity by 50 %. Trolox (6-hydroxy-2,5,7,8-tetramethylchroman-2-carboxylic acid) was used as a drug reference.

#### 2.5.3 Ferric-reducing antioxidant power (FRAP) assay

The FRAP assay was carried out according to the method described in the literature [23]. Briefly, a solution consisting in a 10:1:1 ratio of 23 mM acetate buffer (pH 3.6), 10 mM of tripyridyl triazine (TPTZ) in HCl (40 mM), and FeCl_3_ (20 mM) respectively was prepared as FRAP reagent. The assay was performed in 96 multiwell plate. In each well were placed 264 μL of FRAP reagent and 8 μL of EOs at different concentrations. The reaction mixture was incubated at 37 °C for 30 min in dark conditions. Absorbance was read at 593 nm using a Thermo Scientific Multiskan GO spectrophotometer (Thermo Fischer Scientific, Vantaa, Finland). The absorbance of the blank (FRAP reagent) was subtracted from all absorbances with the sample to determine the FRAP value for each sample. Trolox was used as the drug reference.

#### 2.5.4 β-carotene/linoleic acid bleaching assay

The β-carotene/linoleic acid bleaching assay was carried out by the method described by Miraliakbari and Shahidi, with some modifications [24]. Briefly, 1 mg β-carotene were dissolved in 2 mL chloroform, 50 μL linoleic acid and 400 mg Tween 20. The chloroform was then eliminated by evaporation and 40 mL of distilled water was added to form an emulsion. The assay was performed in 96 multiwell plate, in each well 270 μL of the emulsion and 30 μL of an ethanol solution of EOs to have final concentration of 1 mg/mL, were transferred. All samples were placed at 50 °C in a block heater for 2 h together with a negative control (blank), which contained the same volume of ethanol instead of the samples. Trolox was used as the standard reference.

The absorbance was read at 470 nm with a Thermo Scientific Multiskan GO spectrophotometer (Thermo Fischer Scientific, Vantaa, Finland) at initial time (t = 0) and after 2 h. The capacity of the EOs to protect against oxidation of β-carotene was determined with the following (2), (3):

(2) $$C=\frac{A_{sample\;t=0}-A_{sample\;t=2h}}{A_{control\;t=0}-A_{control\;t=2h}\;}$$

(3) %β−caroteneretention=100%−C*100%

where **C** is carotene depletion factor. A_sample t = 0_ and A_sample t = 2h_ are the absorbance of samples at initial time and after 2 h incubation, respectively. A_control t = 0_ and A_control t = 2h_ are the absorbance values at initial time of the experiments and after 2 h incubation, respectively.

### 2.6 Herbicidal activity of EOs

Phytotoxic activity assays were performed using *Sinapis arvensis*, *Trifolium campestre*, *Lepidium sativum* and *Lolium rigidum* seeds as described by Ben Ghnaya et al. [25] with following modifications. Briefly, the surface of the seeds was sterilized with 95 % ethanol for 10 s to avoid possible contamination. Then the seeds were rinsed with distilled water and put in Petri dishes (Ø = 100 mm), with three Whatman filter paper, imbibed with 7 mL of distilled water for negative control or 7 mL of different doses (8, 6, 4, 2 mg/mL) essential oil dissolved in solution constituted by 95.5 % distillated water and 0.5 % acetone. Cultures were incubated at 20 ± 1 °C, with natural photoperiod. There are no significative differences between controls carried out with water-acetone mixture and controls with water alone. The shoots and roots lengths and the number of germinated seeds were measured after 10 days Each experiment was replicated three times, using Petri dishes containing 10 seeds each. The results are reported as the % of germinated seeds, and as mean ± SD for shoots and radicle elongation.

### 2.7 Anti-enzymatic activity

#### 2.7.1 Cholinesterases inhibition

Cholinesterases inhibition was studied by Ellman's method [26] with following modifications. Four hundred 15 μL of Trizma-HCl buffer 0.1 M (pH 8), 10 μL of different concentrations of EOs dissolved in DMSO solution 1 %, and 25 μL of 0.28 U/mL of AChE or BChE solution were placed for 15 min at 37 °C. Then, 75 μL of a 1.83 mM solution of AChI or BChI and 475 μL of DTNB was added. After 30 min at 37 °C the mix was transferred in 1 mL cuvette and the samples were read at 405 nm in a spectrophotometer (Thermo Fischer Scientific, Vantaa, Finland). Galantamine was the reference drug.

#### 2.7.2 α-Amylase inhibition assay

Amylase activity was studied using the method of Bernfeld [27] with following modifications. A volume of 200 μL of 20 mM phosphate buffer with 6.7 mM NaCl (pH = 6.9), 100 μL of different concentrations of EOs and 100 μL of alpha-amylase water solution (1U/ml) were placed in safe-lock tubes and this mix was placed for 10 min at 37 °C. Then, 180 μL of a starch solution (1 % w/mv) was added, and the reaction was incubated at 37 °C for 10 min. Then, 180 μL of dinitrosalicylic acid (DNSA) reagent (prepared using 20 mL of 3,5-DNSA in distillated water (96 mM), 8 mL of 5.3 M potassium sodium tartrate in 2 M NaOH, and 12 mL of warm distillated water) was added and incubated at 100 °C for 10 min. Samples and control were read at 540 nm with a Thermo Scientific Multiskan GO spectrophotometer (Thermo Fischer Scientific, Vantaa, Finland). In the negative control EOs were replaced with 100 μL of buffer. Acarbose was the reference drug.

#### 2.7.3 α-Glucosidase inhibition assay

Anti- α-Glucosidase activity was determined following the method previously reported [28] with minor modifications. This test was carried out in 96 multiwell plate: 150 μL of 0.1 M phosphate buffer (pH 7.0), 10 μL of the EOs solubilized in methanol to have different concentrations and 15 μL of 1 U/mL α-glucosidase enzyme solution were added to each mix. The plate was placed at 37 °C for 5 min. Then, 75 μL of 2.0 mM 4-nitrophenyl-D-glucopyranoside (pNPG) were added and the plate was placed for 10 min at 37 °C. The absorbance was read at 405 nm using a Thermo Scientific Multiskan GO spectrophotometer (Thermo Fischer Scientific, Vantaa, Finland). Acarbose was the reference drug used as positive control.

The percent inhibition of enzyme activity for cholinesterases, α-amylase and α-glucosidase was calculated by comparison with the absorbance of the control without sample, following formula (4):

(4) $$I\%=\frac{A_{0}-A_{1}}{A_{0}}*100$$

where A_0_ is the absorbance of the control without the sample and A_1_ is the absorbance of the sample. The results were expressed as IC_50_ values, which are the concentrations of EOs necessary to reduce the enzymatic activity by 50 %.

### 2.8 Statistical analysis

All assays were conducted using a randomized block design with 3 replications. Statistical analyses were performed with SPSS 23 Statistical. Results were examined statistically using one-way analysis of variance (ANOVA) followed by SNK tests or by Tukey's post-hoc test. The differences between individual means were considered significant at (p < 0.05).

Multivariate analysis (Hierarchical clustering analysis (HCA) and Principal Coordinates analysis (PCoA) were carried out using PAST 4.13 software.

## 3 Results

### 3.1 Chemical composition

The yields of EOs of were 0.2 %, 0.6 % and 0.3 % for stems, needles and cones*,* respectively. The composition of the EOs is reported in Table 1.

**Table 1:** Chemical composition of the EOs. from *Callitris glaucophylla* stems (A), needles (B) and cones (C).

	A%	B%	C%	KI^a^	KI^b^	I^c^
Tricyclene	0.4	1.8	0.9	844	1047	1,2
α-Pinene	55.5	37.0	59.8	857	1036	1,2,3
Camphene	–	3.9	1.9	866	1075	1,2
Verbenene	–	–	2.3	882		1,2
β-Pinene	3.1	0.8	3.2	894	1136	1,2,3
Myrcene	0.6	13.3	1.1	903	1145	1,2
δ-3-Carene	1.4	1.7	2.2	923	1171	1,2
Limonene	–	18.9	3.0	924	1205	1,2,3
1,8-Cineole	19.9	–	–	1031	1210	1,2,3
γ-Terpinene	–	0.4	–	1059	1221	1,2
Terpinen-4-ol	0.8	0.6	–	1079	1590	1,2,3
α-Terpinolene	0.8	1.6	–	1088		1,2
α-Campholenal	–	–	6.5	1126		1,2
Camphor	–	1.7	–	1133	1491	1,2
Pinocarveol	0.6	–	4.6	1139		1,2
*endo*-Fenchyl acetate	1.3	5.4	0.4	1151		1,2
*p*-Mentha-1,5-dien-8-ol	–	–	2.0	1159	1670	1,2
Verbenone	–	–	3.9	1161		1,2
*cis*-Carveol	–	–	0.8	1176		1,2
Bornyl acetate	1.9	7.9	–	1180	1288	1,2,3
Myrtenal	–	–	1.6	1196	1648	1,2
α-Terpinyl acetate	–	1.2	–	1234	1687	1,2
β-Caryophyllene	1.6	0.9	–	1295	1575	1,2,3
*p*-2,5-Dimethoxy-cymene	–	–	1.5	1304		1,2
α-Humulene	0.9	–	–	1322	1641	1,2
Caryophyllene oxide	1.9	0.7	0.6	1443	2000	1,2
β-Barbatene	0.3			1458		1,2
Valencene	0.7	–	–	1493	1748	1,2
α-Cadinol	1.9	–	–	1507	2256	1,2
Muurolol	1.4	–	–	1645		1,2
Total	94.6	93.1	96.5			
Monoterpenes hydrocarbons	61.0	77.8	74.6			
Oxygenated monoterpenes	25.3	19.5	21.3			
Sesquiterpenes hydrocarbons	3.5	0.9	0			
Oxygenated sesquiterpenes	5.2	0.6	0.6			

^a, b^ The Kovats retention indices are relative to a series of *n*-alkanes (C_10_–C_35_) on the apolar HP-5 MS and the polar HP Innowax capillary columns, respectively.

^c^Identification method: 1 = comparison of the Kovats retention indices with published data, 2 = comparison of mass spectra with those listed in the NIST 02 and Wiley 275 libraries and with published data, and 3 = co-injection with authentic compounds; - = absent.

The phytochemical analysis allowed to identify 31 components representing 93.1 %–96.5 % of the total identified components. All the samples showed a specific richness in monoterpenes hydrocarbons (61.0–77.8 %) and oxygenated monoterpenes (19.5–25.3 %).

Stems samples are mainly composed by monoterpenes hydrocarbons (61.0 %) and oxygenated monoterpenes (25.3 %) and the major compounds identified were α-pinene (55.5 %), 1,8-cineole (19.9 %) and β-pinene (3.1 %). 1,8-cineole, α-cadinol (1.9 %), muurolol (1.4 %) and valencene (0.7 %) were detected only in stem essential oil.

EO from needles was also rich in monoterpene hydrocarbons and oxygenated monoterpenes with percentages of 77.8 and 19.5 %, respectively. Seventeen constituents were identified and α-pinene (37.0 %), limonene (18.9 %), myrcene (13.3 %), bornyl acetate (7.9 %), and *endo*-fenchyl acetate (5.4 %) were the main components.

Seventeen components, representing 96.5 % of the total content, were identified in the cones EO, including 74.6 % monoterpenes hydrocarbons and 21.3 % oxygenated hydrocarbons. The main compounds were: α-pinene (59.8 %), α-campholenal (6.5 %), verbenone (3.9 %), β-pinene (3.2 %), limonene (3.0 %) and pinocarveol (4.6 %).

α-Campholenal, pinocarveol, verbenone, *p*-cymene (2,5-dimethoxy) (1.5 %), *cis*-carveol, (0.8 %), myrtenal (1.6 %), *p*-mentha-1,5-dien-8-ol (2.0 %) and verbenene (2.3 %) characterized only cones EO.

In this study, multivariate analysis was used to describe possible relationships between three essential oils and their chemical compositions, in particular, principal coordinates analysis (PCoA) and a hierarchical cluster analysis (HCA) were carried out.

**Fig. 1:**
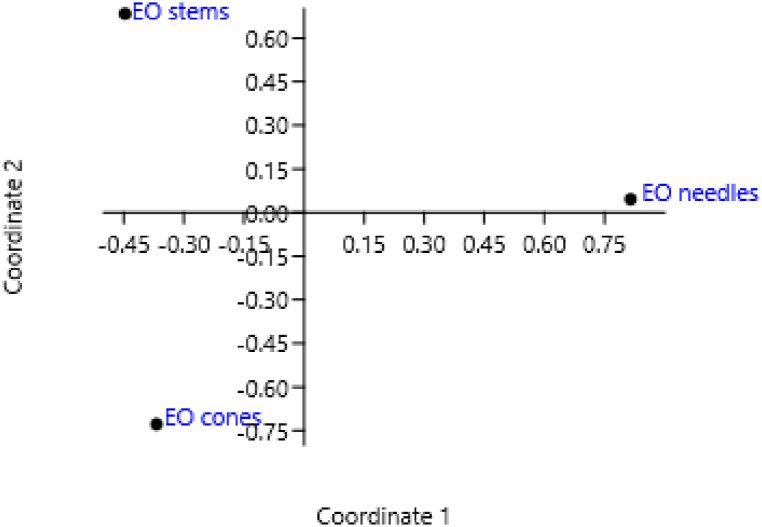
PCoA representation of EOs based on the chemical composition.

The first analysis, the PCoA permitted to simplify data's complexity retaining the information about variability of the variance. As a result of PCoA, 30 essential oils components were sorted into two principal coordinates. These coordinates were represented for each EOs in Fig. 1 and occupied 64.3 % and 35.6 % of the proportion of variance, respectively.

**Fig. 2:**
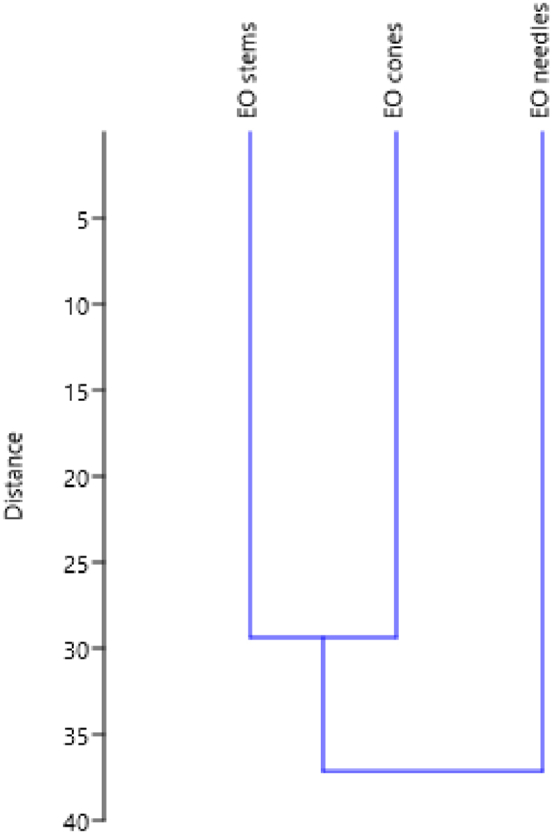
Dendrogram shows three groups of *C. glaucophylla* EOs based on their chemical composition.

The result of hierarchical cluster analysis based on Euclidean correlation coefficient between groups revealed two distinct groups (Group A and Group B), identified by their unique EOs chemotype represented in Fig. 2.

Group A, primarily consisting of needles *C. glaucophylla* EO that stands out as a distinct group in the Principal Coordinates Analysis (PCoA) and exhibits a clear dichotomy in the Hierarchical Cluster Analysis (HCA). The main compound in this EO was α-Pinene but it did not exceed 50 %, moreover this EO contained myrcene, limonene, γ-terpinene, α-terpinolene, camphor, endo-fenchyl acetate, bornyl acetate and α-terpinyl acetate in higher amount respect to the other EOs. Group B was formed by cones and stems *C. glaucophylla* EOs that showed a higher amount of α-Pinene respect to needles *C. glaucophylla* EO and a similar content of compounds previously cited.

### 3.2 Antioxidant activity

The antioxidant capacities of EOs could be affected by some factors, such as the test system, and could not be completely described by one single method. In this study, all three EOs were tested for their free radical scavenging potential by both DPPH and ABTS tests, for their reducing power by FRAP assay, and for their capacity to inhibit to β-carotene oxidation by β-carotene/linoleic acid bleaching assay.

The oils showed antioxidant activities with varying degrees of DPPH inhibition. According to the statistical analyses (p ≤ 0.05), this activity is variable depending on the tested EO. The cones and the needles showed the best DPPH scavenging activities (IC_50_ of 54.15 and 58.69 μg/mL, respectively) followed by the stems (IC_50_ = 76.54 μg/mL), all lower that the standard trolox (IC_50_ = 3.8 μg/mL) (Table 2).

**Table 2:** Antioxidant activities of *C. glaucophylla* EOs.

	DPPH (IC_50_,μg/ml)	ABTS (IC_50_,μg/ml)	FRAP value (mmol Fe (II)/g EO)	% β-carotene retention
Cones	54.2 ± 5.4^a^	15.4 ± 2.1^a^	39.9 ± 3.6^b^	4.3 ± 0.3^a^
Needles	58.7 ± 6.3^a^	13.1 ± 3.5^a^	11.3 ± 2.1^a^	26.8 ± 0.6^b^
Stems	74.5 ± 4.2^b^	26.1 ± 1.8^b^	13.8 ± 1.8^a^	n.a
Trolox	3.8 ± 0.2	5.8 ± 0.3	5.2 ± 0.4	94.8 ± 3.1

Data are the means ± standard deviation of three experiments. Trolox is used as reference standard in antioxidant assays. Means followed by different letters in the same column indicate that are significantly different at p < 0.05, according to a two-way ANOVA followed by Tukey's post hoc test.

In terms of ABTS free radical scavenging activity, significant variability depending on the part of the plant was recorded. The best activities were obtained with the needles and cones EOs (IC_50_ = 13.14 and 15.39 μg/mL respectively), twice the activity of the stems EO (IC_50_ = 26.06 μg/mL). These activities remain even lower if compared with Trolox used as antioxidant reference drug which showed an IC_50_ value of 5.8 μg/mL (Table 2).

According to the FRAP method the EO from cones showed the major ability to reduce ferric iron with a F value of 39.9 mmol Fe(II)/g EO. The other two samples showed a similar activ-ity with FRAP values of 11.3 and 13.8 mmol Fe(II)/g EO, respectively for the EO from nee-dles and stems (Table 2). However, the activities of EOs were higher than Trolox that showed a FRAP value of 5.2 mmol/g.

For that concern EOs capacity to inhibit to β-carotene oxidation, the data showed that EO derived from needles showed the main activity with an inhibition of 26.8 % of β-carotene oxidation respect to EO from cones and stems (Table 2). However, needles EO activity is three time less than Trolox used as antioxidant standard.

### 3.3 Phytotoxic activity

*C. glaucophylla* EOs have been tested for their phytotoxic effects on the germination and growth of aerial parts and roots of *S. arvensis*, *T. campestre* (dicot weeds), *L. rigidum* (a monocot weed) and *L. sativum* as a monocot crop. The phytotoxicity of EOs was evaluated in comparison with a negative control and the activity of two synthetic herbicides as positive controls (pendimethalin and glyphosate). The results are shown in Table 3, Table 4, Table 5. Moreover, in Table 6 was reported IC_50_ values of phytotoxic effects against germination, shoots and roots growth. A significant herbicidal effect which depended on the EO (needles, cones and stems), the doses and the herbs tested was registered.

**Table 3:** Phytotoxic activity of the essential oils of *C. glaucophylla* against germination of *S. arvensis, L. rigidum, T. campestre, L. sativum*. Results are expressed as the mean of three experiments ± standard deviation.

		Germination (%)			Control+	
	Doses (mg/mL)	Needles	Stems	Cones	Pendimethalin	Glyphosate
*S. arvensis*	C-	96.66 ± 0.57Aa	96.66 ± 0.57Aa	96.66 ± 0.57Aa	96.66 ± 0.57^Aa^	96.66 ± .57^Aa^
	2	26.3 ± 0.57 Bb	6.66 ± 0.57 Bb	16.66 ± 0.57^Bb^	100± 0^Aa^	26.66 ± 2.51^Bb^
	4	20 ± 0 BCb	0 ± 0 Bc	0 ± 0 Cc	93.33 ± 0.57 Aa	23.33 ± 0.57 Bb
	6	13.33 ± 0.57 Cb	0 ± 0 Bc	0 ± 0 Cc	93.33 ± 0.57 Aa	0 ± 0 Bc
	8	0 ± 0 Db	0 ± 0 Bb	0 ± 0 Cc	73.33 ± 0.57 Ba	0± 0Bb
*L. rigidum*	C-	93.33 ± 0.57 Aa	93.33 ± 0.57 Aa	93.33 ± 0.57Aa	93.33 ± 0.57 Aa	93.33 ± 0.57 Aa
	2	50 ± 0 Bc	50 ± 0 Bc	93.33 ± 0.57Aa	13.33 ± 1.15 Bd	80± 1Bb
	4	10 ± 0 Cc	13 ± 0.57 Cc	80 ± 0 Ca	0 ± 0 Bd	50 ± 1 Cb
	6	10 ± 0 Cb	10 ± 0 Cb	20 ± 0 Ca	0 ± 0 Bc	0 ± 0 Dc
	8	0 ± 0 Da	0 ± 0 Da	0 ± 0 Da	0 ± 0 Ba	0 ± 0 Da
*T. campestre*	C-	100 ± 0 Aa	100 ± 0 Aa	100 ± 0 Aa	100 ± 0 Aa	100 ± 0 Aa
	2	86.6 ± 0.57 ABa	90 ± 0 Ba	90 ± 0 Ba	83.33 ± 0.57 Ba	80 ± 1 Bb
	4	90 ± 0 Aba	83.33 ± 0.57 BCa	83.33 ± 0.57Ca	83.33 ± 0.57 Ba	50± 1Cb
	6	80 ± 0 Ba	83.33 ± 0.57 BCa	0 ± 0 Db	83.33 ± 0.57 Ba	0 ± 0 Db
	8	93.33 ± 0.57 ABa	80 ± 0 Ca	0 ± 0 Dd	63.33 ± 0.57 Cb	0 ± 0 Db
*L. sativum*	C-	100 ± 0 Aa	100 ± 0 Aa	100 ± 0 Aa	100 ± 0 Aa	100 ± 0 Aa
	2	100 ± 0 Aa	93.33 ± 0.57 Aa	100 ± 0 Aa	86.66 ± 0.57 Aa	36.66 ± 0.57 Bb
	4	86.66 ± 0.57 BCa	73.33 ± 0.57 Bb	66.66 ± 0.57Bc	83.33 ± 0.57 Aa	0 ± 0 Cd
	6	93.33 ± 0.57 ABa	30 ± 0 Cc	0 ± 0 Cd	83.33 ± 0.57 Ab	0 ± 0 Cd
	8	83.33 ± 0.57 Ca	0 ± 0 Db	0 ± 0 Cb	83.33 ± 0.57 Aa	0 ± 0 Cb

Means of the same column by the same uppercase letter and means in the same line by the same lowercase are not significantly different according Student-Newman-Keuls test at (p ≤ 0.05). C-: negative control. C+: positive controls.

**Table 4:** Phytotoxic activity of the essential oils of *C. glaucophylla* against shoots growth (cm)of *S. arvensis, L. rigidum, T. campestre, L. sativum*. Results are expressed as the mean of three experiments ± standard deviation.

		Shoots growth			C+	
	Doses (mg/mL)	Needles	Stems	Cones	Pendimethalin	Glyphosate
*S. arvensis*	C-	2.95 ± 0.17 A	2.95 ± 0.17 A	2.95 ± 0.17 A	2.95 ± 0.17 A	2.95 ± 0.17 A
	2	1.19 ± 0.04 ab	0.33 ± 0.28Bc	1.5 ± 0.28Ba	1.00 ± 0.02 Bb	0.5 ± 0.1 Bc
	4	0.83 ± 0.14Cb	00 ± 00 Cd	00 ± 00 Cd	1.00 ± 0.04 Aa	0.26 ± 0.12ACc
	6	0.83 ± 0.14 Ca	0 ± 0 Cb	0 ± 0 Cb	0.93 ± 0.01 Aa	0 ± 0 Cb
	8	0 ± 0 Db	0 ± 0 Cb	0 ± 0 Cb	0.56 ± 0.10 Ca	0 ± 0 Cb
*L. rigidum*	C-	6.14 ± 0.73 A	6.14 ± 0 .73 A	6.14 ± 0.73 A	6.14 ± 0.73 A	6.14 ± 0.73 A
	2	2.40 ± 0.34Bb	2.41 ± 0.42Bb	6.63 ± 0.61Ba	0.25 ± 0.20 Bb	0 ± 0 Bc
	4	1.33 ± 0.28Cb	1.02 ± 0.31Cb	5.62 ± 0.43Ba	0.23 ± 0.20 Cc	0 ± 0 Bb
	6	0.66 ± 0.28Cb	0 ± 0 Dc	2.58 ± 0.14Ca	0 ± 0 Cc	0 ± 0 Bc
	8	0 ± 0 Da	0 ± 0 Da	0 ± 0 Da	0 ± 0 Ca	0 ± 0 Ba
*T. campestre*	C-	1.67 ± 0.14 A	1.67 ± 0.14 A	1.67 ± 0.14 A	1.67 ± 0.14 A	1.67 ± 0.14 A
	2	1.15 ± 0.13 Ba	1.09 ± 0.12 Ba	0.60 ± 0.07 Bb	0.40 ± 0.03 Bc	0.18 ± 0.01 Bc
	4	0.48 ± 0.01 Cb	0.82 ± 0.13 BCa	0.50 ± 0 Bb	0.31 ± 0.01 Bc	0.15 ± 0.01 Bd
	6	0 ± 0 Dd	0.69 ± 0 .12 BCa	0.54 ± 0.07 Bb	0.37 ± 0.05 Bc	0 ± 0 Cd
	8	0 ± 0 Dd	0.82 ± 0.12 BCa	0.56 ± 0.05 Bb	0.27 ± 0.12 Bc	0 ± 0 Cd
*L. sativum*	C-	1.76 ± 0.23 A	1.76 ± 0.23 A	1.76 ± 0.23 A	1.76 ± 0.23 A	1.76 ± 0.23 A
	2	0.90 ± 0.04 Bb	1.30 ± 0.17 Ba	1.57 ± 0.35 Ba	0.62 ± 0.01 Bb	0.27 ± 0.23 Bc
	4	0.56 ± 0.18 Cb	0.93 ± 0.25 Ca	0.48 ± 0.09 Cb	0.64 ± 0.05 Bb	0.15 ± 0.04 BCc
	6	0.43 ± 0.03 Cb	0.31 ± 0.07 Dc	0 ± 0 Dd	0.54 ± 0.10 Ba	0 ± 0 Cd
	8	0 ± 0 Db	0 ± 0 Db	0 ± 0 Db	0.36 ± 0.02 Ba	0 ± 0 Cb

Means of the same column by the same uppercase letter and means in the same line by the same lowercase are not significantly different according Student-Newman-Keuls test at (p ≤ 0.05). C-: negative control. C+: positive controls.

**Table 5:** Phytotoxic activity of the essential oils of *C. glaucophylla* against roots growth (cm) of *S. arvensis, L. rigidum, T. campestre, L. sativum*. Results are expressed as the mean of three experiments ± standard deviation.

		Roots growth			C+	
	Doses (mg/mL)	Needles	Stems	Cones	Pendimethalin	Glyphosate
*S. arvensis*	C-	5.82 ± 0.81 A	5.82 ± 0.81 A	5.82 ± 0.81 A	5.82 ± 0.81 A	5.82 ± 0.81 A
	2	1.58 ± 0.14 Bc	0.33 ± 0.28 Bd	2.26 ± 0.28 Bb	3.05 ± 0.26 Ba	0.56 ± 0.25 Bd
	4	1.75 ± 0.43 Ba	0 ± 0 Bc	0 ± 0 Cc	1.77 ± 0.14 Ca	0.5 ± 0 Bb
	6	0.83 ± 0.28 Ba	0 ± 0 Bc	0 ± 0 Cc	1.18 ± 0.08 DEab	0 ± 0 Bc
	8	0 ± 0 Cb	0 ± 0 Bb	0 ± 0 Cb	0.66 ± 0.04 Ea	0 ± 0 Bb
*L. rigidum*	C-	6.78 ± 0.1 A	6.78 ± 0.1 A	6.78 ± 0.1 A	6.78 ± 0.1 A	6.78 ± 0.1 A
	2	2.56 ± 0.11 Bb	2.8 ± 0.04 Bb	6.87 ± 0.65 Aa	0.66 ± 0.57 Bc	0 ± 0 Bc
	4	1.5 ± 0.86 Cb	1.5 ± 0.13 Cb	5.27 ± 0.18 Ba	0 ± 0 Cc	0 ± 0 Bc
	6	1 ± 0 Cb	0 ± 0 Dc	1.91 ± 0.14 Ca	0 ± 0 Cc	0 ± 0 Bc
	8	0 ± 0 D	0 ± 0 D	0 ± 0 D	0 ± 0C	0 ± 0 B
*T. campestre*	C-	4.53 ± 0.54 A	4.53 ± 0.54 A	4.53 ± 0.54 A	4.53 ± 0.54 A	4.53 ± 0.54 A
	2	3.22 ± 0.48 Ba	3.4 ± 0.38 Aa	1.94 ± 0.26 Bb	0.46 ± 0.15 Bc	0.58 ± 0.06 Bc
	4	2.61 ± 0.09 Ca	2.77 ± 0.04 ABb	0.96 ± 0.21 Cd	0.53 ± 0 Bc	0.47 ± 0.02 Bd
	6	1.34 ± 0.1 Cab	2.13 ± 0.5 Ba	0 ± 0 Db	0.59 ± 0.02 Bb	0 ± 0 Bb
	8	0.5 ± 0 Da	1.31 ± 0.13 Cb	0 ± 0 Dd	0.45 ± 0.01 Bc	0 ± 0 Bd
*L. sativum*	C-	10.01 ± 0.7 A	10.01 ± 0.7 A	10.01 ± 0.7 A	10.01 ± 0.7 A	10.01 ± 0.7 A
	2	2.02 ± 0.4 Bbc	1.2 ± 0.17 Bc	4.74 ± 1.6 Ba	3.05 ± 0.26 Bb	0.62 ± 0.06 Bc
	4	1.7 ± 0.38 Ba	0.7 ± 0.25 BCb	0.82 ± 0.07 Cb	1.77 ± 0.14 Cab	0.42 ± 0.02 Bb
	6	1.34 ± 0.08 Ba	0 ± 0 Cc	0 ± 0 Cc	1.18 ± 0.08 CDb	0 ± 0 Bc
	8	0.75 ± 0.1 Ca	0 ± 0 Cb	0 ± 0 Cb	0.66 ± 0.04 Da	0 ± 0 Bb

Means of the same column by the same uppercase letter and means in the same line by the same lowercase are not significantly different according Student-Newman-Keuls test at (p ≤ 0.05). C-: negative control. C+: positive controls.

**Table 6:** IC_50_ of phytotoxic effects against germination, shoots and roots growth.

	IC_50_ (mg/ml)		
	Germination	Shoots growth	Roots growth
	*S. arvensis*		
Needles	1.3 ± 0.5^a^	1.7 ± 0.4^ab^	1.3 ± 0.1^a^
Stems	1.0 ± 0.2^a^	1.1 ± 0.2^a^	1.1 ± 0.2^a^
Cones	1.2 ± 0.3^a^	2.1 ± 0.3^b^	1.6 ± 0.6^a^
Pendimethalin	n.a	1.5 ± 0.1^a^	1.1 ± 0.7^a^
Glyphosate	1.3 ± 0.1^a^	4.8 ± 0.4^c^	2.1 ± 0.4^b^
	*L. rigidum*		
Needles	2.0 ± 0.3^a^	1.6 ± 0.3^a^	1.6 ± 0.5^a^
Stems	2.1 ± 0.7^a^	1.6 ± 0.2^a^	1.7 ± 0.4^a^
Cones	4.3 ± 0.5^b^	5.2 ± 0.7^b^	4.8 ± 0.7^b^
Pendimethalin	1.2 ± 0.4^a^	1.0 ± 0.7^a^	1.1 ± 0.1^a^
Glyphosate	4.0 ± 0.2^b^	1.0 ± 0.7^a^	1.0 ± 0.7^a^
	*T. campestre*		
Needles	n.a	1.4 ± 0.2^a^	4.2 ± 0.4^b^
Stems	n.a	2.2 ± 0.5^a^	5.7 ± 0.3^c^
Cones	5.0 ± 0.7^a^	1.6 ± 0.3^a^	1.7 ± 0.1^a^
Pendimethalin	n.a	1.3 ± 0.4^a^	1.1 ± 0.2^a^
Glyphosate	4.0 ± 0.5^a^	1.1 ± 0.6^a^	1.1 ± 0.3^a^
	*L. sativum*		
Needles	n.a	0.9 ± 0.5^a^	1.2 ± 0.5^a^
Stems	4.8 ± 0.3	4.1 ± 0.3^c^	1.1 ± 0.6^a^
Cones	5.8 ± 0.4	2.7 ± 0.4^b^	1.9 ± 0.3^a^
Pendimethalin	n.a	1.5 ± 0.6^ab^	1.4 ± 0.2^a^
Glyphosate	1.6 ± 0.2	1.2 ± 0.7^a^	1.1 ± 0.5^a^

Means followed by different letters in the same column indicate that are significantly different at p < 0.05, according to a one-way ANOVA followed by Tukey's post hoc test. n.a = IC_50_ > 8 mg/mL

For *S. arvensis*, a total inhibition of germination was observed at 2 mg/mL of cones and stems EOs, and at 8 mg/mL for needles EO. Similarly, a total inhibition for glyphosate was recorded at 6 mg/mL, whereas pendimethalin showed moderate herbicidal activity (germination was reduced only to 73.33 %) even at high doses (8 mg/mL). The IC_50_ values showed that *S. arvensis* seeds germination was inhibited in similar way by three EOs respect to glyphosate, instead for shoots growth only needles and stems EOs were like pendimethalin. For that concern roots growth, the EOs results are comparable to the pendimethalin.

A total inhibition of *L. rigidum* germination was obtained at 6 μL/mL for pendimethalin and at 8 mg/mL for cone, needle and stem EOs whereas glyphosate allowed total inhibition at a lower dose (4 μL/mL). IC_50_ values showed that *L. rigidum* shoots and roots growth were inhibited by needles and stems EOs at similar concentration of both positive controls. Instead, *L. rigidum* germination was inhibited by needles and stems like pendimethalin and by cones EO like glyphosate.*T. campestre* and *L. sativum* showed resistance to needle and stem EOs and to pendimethalin, while total inhibition of germination was obtained with cone EO and glyphosate at 6 mg/mL for *T. campestre* and 6 and 8 mg/mL for *L. sativum* using cone and stems EOs, respectively.

For that concern IC_50_ values for *T. campestre* seeds shoots growth all EOs and positive controls showed a similar activity, but only EO from cones was active against germination and its activity is like that of glyphosate. The cones EO was also the most active against *T. campestre* roots growth and comparable to that of pendimethalin and glyphosate.

In L. *sativum* seeds roots growth was inhibited by EOs and both positive controls in same way. Needle EO was the most active against shoots growth with IC_50_ value comparable to both positive controls instead stems and cones EOs were active against *L. sativum* germination but less respect to glyphosate.

Generally, the antigerminative activity of the EOs is significant, comparable to that of the two reference herbicides, variable depending on doses and EOs and tested herbs.

All the EOs inhibited the growth of shoots and roots of tested species in a dose-dependent manner (p ≤ 0.05). According to statistical analysis, the growth inhibition effects are remarkable at low and high doses. Growth inhibition is also variable depending on the oil and tested species. Likewise, the inhibition effects are comparable to those of the reference herbicides.

### 3.4 Anti enzymatic activities

The anti-enzymatic activities are reported in Table 7. The cones EO was the most active against AChE (IC_50_ 89.8 μg/mL), respect to the EOs derived from needles and stems. Instead, the needles EO showed major activity against BChE respect to the others EOs with an IC_50_ value of 172.0 μg/mL. For both enzymes the stems EO showed the lower activity. The IC_50_ value of all EOs was higher than galantamine used as positive control. On the other hand, the stems EO is more active against α-amylase and α-glucosidase respect to EOs from cones and needles with IC_50_ of 4096.0 μg/mL and 7402.1 μg/mL, respectively for α-amylase and α-glucosidase. The samples showed less activity respect to acarbose used as positive control.

**Table 7:** Inhibitory effects of the EOs on AChE. BChE. α -amylase and α-glucosidase.

	IC_50_ (μg/ml)			
	AChE	BChE	α-amylase	α-glucosidase
Cones	89.8 ± 1.8	220.1 ± 4.6	n.a	n.a
Needles	117.1 ± 1.0	172.0 ± 2.4	n.a	n.a
Stems	227.7 ± 5.0	6575.5 ± 83.7	4096.0 ± 312.0*	7402.1 ± 152.0
Galantamine	0.9 ± 0.4	4.6 ± 1.5	–	–
Acarbose	–	–	12.0 ± 3.2	973.0 ± 0.32

Data are the means ± standard deviation of three experiments. Means followed by different letters in the same column indicate that are significantly different at p < 0.05, according to a two-way ANOVA followed by Tukey's post hoc test. n.a = IC_50_ > 10,000 μg/mL ^.^.

## 4 Discussion

### 4.1 Chemical composition

The study carried out by Brophy and coworkers [29] on the chemical composition of Australian EOs from the needles of 18 species and four subspecies of *Callitris* genu*s* including *C. glaucophylla* revealed that α-pinene (29–30.2 %), limonene (23.4–26.4 %), myrcene (8.9–19 %), α-fenchyl acetate (7–8.5 %) and bornyl acetate (4.8–7%) were the major components: these results corroborated with those obtained in the present research.

In another study conducted in Australia in 2014 on the EOs of *C. glaucophylla* needles, similar results were obtained and showed the richness of the analyzed EOs in limonene (23.4–26.4 %), α-pinene (17.4–22.8 %), myrcene (22.2–26.2 %), α-fenchyl acetate (11.7–19.7 %) and bornyl acetate (16.1 %) [30].

However, there is no study reported in the literature on the composition of cones and stems EOs. The present study is the first investigation, and it can provide additional data on the chemistry of *C. glaucophylla.* The presence of 1,8-cineole in the stems oil sample has been also mentioned in *Tetraclinis articulata* (Vahl) Mast. EO (Cupressaceae) [8].

The variation in yields and EOs composition from the different parts of plant have been reported in the literature, in particular in the Cupressaceae family. A recent study on EOs produced by *Cupressus arizonica* Greene revealed qualitative and quantitative variability in EOs among plant organs (cones, leaves and stems) [7]. Our study showed similar results with the literature for oils obtained from *Callitris glaucophylla* needles growing widely in Australia. These results confirm the adaptation and acclimatization of the species to pedoclimatic conditions. The Tunisian needles oil showed the same chemotype of the species grown in Australia with some differences that may be related to several factors, in particular sampling, extraction conditions and the different environmental conditions in the two countries [4].

### 4.2 Antioxidant activity

The variability showed in antioxidant results can be due to the diversity of the chemical compositions of the three EOs, knowing that EOs of *C. glaucophylla* were not studied for their antioxidant activity, but the EOs from species of the Cupressaceae family were reported for their significant antioxidant potential [31,32].

In a recent study, Khammassi and coworkers, studied the antioxidant activity of EOs and extracts obtained from needles of *Biota orientalis* (L.) Endl. According to this study, the EOs showed inhibitory activities of DPPH, ABTS, FRAP and a significant total antioxidant capacity [31].

In another report, the EOs from two Cupressaceae, *Juniperus macrocarpa* Sm. and *Juniperus oxycedrus* L.*,* exhibited a significant antioxidant activity when tested by several methods (DPPH, ABTS, FRAP and β-carotene-bleaching test) [32]. Similarly, an antioxidant assay was conducted on the leaves of *Juniperus communis* L., that showed an antioxidant effect of 65 % when compared to ascorbic acid [33]. EOs are a mixture of compounds, and the antioxidant potential depends on the major compounds and the synergism and antagonisms between oil components. The EOs of *C. glaucophylla* have shown a richness in monoterpene hydrocarbons. Limonene, α- and β-pinene, among their main components, have been reported for their antioxidant activity [34,35]. According to these studies, the Ferric reducing power, DPPH and ABTS radical scavenging activity of these compounds exceeds that of α-tocopherol [35] but were lower than that of ascorbic and caffeic acids [34]. Oxygenated monoterpenes also are known for having antioxidant potential [36,37]. Moreover, the high amount of this fraction may explain the antioxidant activities observed in our study. According to the literature, the antioxidant potential is generally related to the presence of compounds derived from *p*-cymene (like thymol and carvacrol), while this class of compounds is absent in *C. glaucophylla* oils. However, the antioxidant potential of several terpenes has been reported, especially for terpenes with conjugated double bonds which neutralize the DPPH and ABTS radicals, which is associated with the loss of the allylic hydrogen atom. This could explain the activities observed in this study [36,37].

#### 4.2.1 Phytotoxic activity

The phytotoxic activity observed in this study are related to the allelopathic potential of *C. glaucophylla* metabolites, in particular of its volatile fraction. These results agree with the literature; indeed, several forest species have shown similar results. Likewise, the major species of the Cupressaceae family are known to have phytotoxic activities against the germination and growth of weeds [3,8]. In recent studies, the EOs of *Juniperus oxycedrus* and *J. sphoniceae* characterized by their richness in pinene (42–49 %), were tested against the germination and growth of monocots and dicot weeds (*Sinapis arvensis*, *Trifolium campestre*, *Lolium rigidum*, *Phalaris canariensis* and *P. paradoxa*) [3,38]. These EOs reduced number of germinated seeds and growth of tested weeds and induced oxidative stress resulting in an alteration of roots membrane integrity [38]. The results obtained in this study confirmed other researches that demonstrated that the EOs of *Cupressus sempervirens* L. var. *dupreziana* (A. Camus) Silba [6] and *Tetraclinus articulata* [25], two Cupressacae, showed a clear herbicidal activity on the germination and growth of *Sinapis arvensis* and *Phalaris canariensis* and wheat [6,25]. The variability of the phytotoxic potential of EOs depending on the part of the plant (needles, cones and stems) has been previously described for species of Cupressacae [2,39]. Several studies have been carried out on the herbicidal potential of the EOs of the different parts of *Pinus pinaster* Aiton (needles, leaves and cones) [39] and *Biota orientalis* (cones and needles) [2]. These researches revealed significant variability and a variable phytotoxic potential depending on the plant part, which was explained by the variability in the chemical composition of the EOs produced by each part of the plant [2,39]. Similarly, in agreement with our data, the majority of studies have described a selectivity of the herbicidal activity of EOs according to weeds species. In fact, the herbicidal potential varies according to the species tested (mono/dicots) and also weeds/cultivated crops [39]. Some of the constituents of the EOs have been reported for their phytotoxic activity; in general, the tested EOs were characterized by their richness in monoterpene hydrocarbons (61.0–77.8 %) and oxygenated monoterpenes (19.5–25.3 %) which are known to have phytotoxic effects [ 40, 41,42]. According to the results presented in Table 3, Table 4, Table 5 and in agreement with the work of De Martino et al., α-pinene (36.9–59.8 %), camphene (0–3.9 %), β-pinene (0.8–3.2 %), myrcene (0.6–13.3 %), limonene (0–18.9 %), 1,8-cineole (19.9 % in stems EO), γ-terpinene (0.4 % in needles EO) and camphor (1.7 % in needles EO) are compounds known for their antigerminative effects against at least *Lepidium sativum* and *Raphanus sativus* [40]. This could explain the phytotoxic effects of *C. glaucophylla* EOs. Moreover, α-pinene, myrcene, limonene and 1,8-cineole have been tested on the inhibition of germination and growth of *Amaranthus retroflexus* L., *Chenopodium album* L. and *Rumex crispus* L. seedlings with different degrees of phytotoxicity, being oxygenated monoterpenes more effective than hydrocarbons [41]. On the other hand, 1,8-cineole showed high degrees of phytotoxicity compared to limonene, cymene and α-pinene and weaker when compared with other oxygenated monoterpenes, notably camphor, terpinen-4-ol which characterize *C. glaucophylla* oils [41]. Sesquiterpenes and sequiterpenoids may contribute to the herbicidal activity.

The variability of activity recorded between the three EOs and even the selectivity of susceptibility of tested herbs, can be the results of a synergism or an antagonism between the EOs constituents. These processes are important and play a positive role in describing EOs as herbicide candidates and able of solving, at least in part, the problems of weeds resistance posed by chemical herbicides. Indeed, it is possible that the different EOs components can act at the same time but with different modes of action in relation to their different chemical classes, structures, polarity, chemical functions. In fact, several modes of action of EOs and their major compound was reported [42,43]. Recent studies indicate that the action of the EOs are related to their apolar nature, which could facilitate their penetration through cell membranes, altering membrane integrity [44,45]. Other studies indicate that terpenes are able to cross the cell to interact with the plant plasma membrane [42,44,46]. α-Pinene, the main components of *C. glaucophylla* EOs, inhibitedthe growth of several plants by altering membrane integrity by the increase in the rate of malondialdehyde which is a product resulting from the lipid peroxidation of membrane phospholipids [47]. This was also confirmed by a study on the EOs of *Juniperus oxycedrus* [38]. Similarly, α-pinene has been described to induce the production of reactive oxygen species and also to stimulate the oxidative defense system of the plant by activation of superoxide dismutase, glutathione reductase peroxidase and guaiacol peroxidase. In addition, this compound, due to its apolar nature, has been reported to induce an alteration in the energy metabolism of plants, by the uncoupling between the oxidative phosphorylation reactions and the synthesis of ATP and inhibiting the electron transport chain [48]. Similarly, the three monoterpenes 1,8-cineole, α-pinene and limonene, major components of *C. glaucophylla* EOs, were tested on mitochondria isolated from maize roots resulting in an inhibition of respiration [49]. 1,8-Cineole was described for inhibiting growth of *Echinochloa crus-galli* (L.) P. Beauv. and *Cassia obtusifolia* L. and mitosis in onion roots in its different phases [50]. Moreover, this monoterpene induced swelling of the root by an alteration of the cortical microtubules in onion seedling [43]. If the volatility of EOs could be an advantage for their use as herbicides in fact the residual of EO compounds in aliments and environment will be near to zero, on the other hand, the same volatility could induce to use a big quantity of EOs which could negatively affect the sphere. A possible solution can be encapsulation that permits to preserve activity as a herbicide without to damage the environment [51].

#### 4.2.2 Anti-enzymatic activities

Some species from Cupressaceae family, among them *C. glaucophylla*, *C. sulcata* (Parl.) Schltr. and *Neocallitropsis pancheri* (Carriére) de Laub. Have been reported for their activity against different mosquito species such as *Aedes aegypti* Linnaeus, 1762 and *Culex annulirostris* Skuse, 1889 and they were resistant to insect attack [16,52]. Some insecticides exert their toxicity by inhibiting the enzyme acetylcholinesterase (AChE) an essential neurotransmitter which degrades acetylcholine (AChE) [53]. Moreover, anti-AChE and anti-BChE activity indicated insecticidal potentialities [54], but nowadays, no studies are present in the literature about the possible effect of *C. glaucophylla* EOs against cholinesterases, even if the research on possible use of extracts from plants of Cupressaceae as disinfestant are developing [16,52]. α-Amylase is a key enzyme that hydrolyses the starch reserves of seeds during germination process, forming maltose from starch [55]; α-glucosidase converts maltose to glucose playing an important role in seed growth regulation [56]. The anti-α-amylase and anti-α-glucosidase activities confirmed the data of phytotoxic activity: in fact, the stems EO was more active at lowest concentration, in particular, against *S. arvensis* seeds germination, in fact at dose of 2 mg/mL germinated only 6.66 % of seeds instead after treatment with a same dose of needles and cones EOs germinated 26.3 % and 16.6 % of seeds respectively. Moreover, stems EO was more active at lowest concentration (2 mg/mL) against shoots growth of *S. arvensis* seeds in fact the shoots length of seeds was 0.33 cm *vs* 1.19 cm for seeds trated with needles EO and 1.5 cm for seeds treated with cones EO. and roots growth of *S. arvensis, L. sativum,* espect to the EOs from needles and from cones.

Stems EO was also more active at lowest concentration (2 mg/mL) against roots growth of *S. arvensis* and *L. sativum* seeds in fact *S. arvensis* roots length of seeds was 0.33 cm *vs* 1.58 cm for seeds treated with needles EO and 2.26 cm for seeds treated with cones EO; while *L. sativum* roots length of seeds was 1.2 cm *vs* 2.02 cm for seeds treated with needles EO and 4.74 cm for seeds treated with cones EO.

Previously, nostudies reported data about the activity of *C. glaucophylla* EOs against α-amylase and α-glucosidase activity.

## 5 Conclusion

This present study is a contribution to the identification of the composition of the volatile fractions of *C. glaucophylla* grown in Tunisia, especially for the cones and the stems, studies for the first time, and a description of the antioxidants, anti-enzymatic and herbicide activity of its EOs. The results of this research can give a glimpse of developments: the antioxidant, anti-enzymatic and especially herbicidal potential makes these EOs possible candidates for herbicides and insecticide agrochemicals, also considering that their activity exceeds that of synthetic herbicides and can contribute to solve the problem of acquisition of resistance to chemical herbicides by weed populations.

## Data availability statement

Data will be made available on request.

## CRediT authorship contribution statement

**Oumayma Kochti:** Methodology, Investigation, Data curation. **Flavio Polito:** Writing - review & editing, Methodology, Investigation, Data curation. **Lucia Caputo:** Writing - original draft, Methodology, Investigation, Data curation. **Khammassi Marwa:** Methodology, Investigation, Data curation. **Yassine Mabrouk:** Writing - original draft, Methodology, Investigation, Data curation. **Lamia Hamrouni:** Writing - original draft, Methodology, Formal analysis, Data curation. **Ismail Amri:** Writing - review & editing, Supervision, Data curation, Conceptualization. **Vincenzo De Feo:** Writing - review & editing, Writing - original draft, Supervision, Conceptualization.

## Declaration of competing interest

The authors declare that they have no known competing financial interests or personal relationships that could have appeared to influence the work reported in this paper.
